# Decision Making under Uncertainty: A Quasimetric Approach

**DOI:** 10.1371/journal.pone.0083411

**Published:** 2013-12-20

**Authors:** Steve N'Guyen, Clément Moulin-Frier, Jacques Droulez

**Affiliations:** Laboratoire de Physiologie de la Perception et de l'Action, UMR 7152 Collège de France - CNRS, Paris, France; Universita' degli Studi di Milano (University of Milan), Italy

## Abstract

We propose a new approach for solving a class of discrete decision making problems under uncertainty with positive cost. This issue concerns multiple and diverse fields such as engineering, economics, artificial intelligence, cognitive science and many others. Basically, an agent has to choose a single or series of actions from a set of options, without knowing for sure their consequences. Schematically, two main approaches have been followed: either the agent learns which option is the correct one to choose in a given situation by trial and error, or the agent already has some knowledge on the possible consequences of his decisions; this knowledge being generally expressed as a conditional probability distribution. In the latter case, several optimal or suboptimal methods have been proposed to exploit this uncertain knowledge in various contexts. In this work, we propose following a different approach, based on the geometric intuition of distance. More precisely, we define a goal independent quasimetric structure on the state space, taking into account both cost function and transition probability. We then compare precision and computation time with classical approaches.

## Introduction

It's Friday evening, and you are in a hurry to get home after a hard day's work. Several options are available. You can hail a taxi, but it's costly and you're worried about traffic jams, common at this time of day. Or you might go on foot, but it's slow and tiring. Moreover, the weather forecast predicted rain, and of course you forgot your umbrella. In the end you decide to take the subway, but unfortunately, you have to wait half an hour for the train at the connecting station due to a technical incident.

Situations like this one are typical in everyday life. It is also undoubtedly a problem encountered in logistics and control. The initial state and the goal are known (precisely or according to a probability distribution). The agent has to make a series of decisions about the best transport means, taking into account both uncertainty and cost. This is what we call *optimal control under uncertainty*.

Note that he might also have an intuitive notion of some abstract distance: how far am I from home? To what extent will it be difficult or time consuming to take a given path? The problem might become even more difficult if you do not know precisely what state you are in. For instance, you might be caught in a traffic jam in a completely unknown neighborhood.

This problem that we propose to deal with in this paper can be viewed as sequential decision making, usually expressed as a Markovian Decision Process (MDP) [1]–[4] and its extension to Partially Observable cases (POMDP) [5], [6]. Knowing the transition probability of switching from one state to another by performing a particular action as well as the associated instantaneous cost, the aim is to define an optimal policy, either deterministic or probabilistic, which maps the state space to the action state in order to minimize the mean cumulative cost from the initial state to a goal (goal-oriented MDPs).

This class of problems is usually solved by Dynamic Programming method, using Value Iteration (VI) or Policy Iteration (PI) algorithms and their numerous refinements. Contrasting with this model-based approach, various learning algorithms have also been proposed to progressively build either a value function, a policy, or both, from trial to trial. Reinforcement learning is the most widely used, especially when transition probabilities and cost function are unknown (model-free case), but it suffers from the same tractability problem [7]. Moreover one significant drawback to these approaches is that they do not take advantage of the preliminary knowledge of cost function and transition probability.

MDPs have generated a substantial amount of work in engineering, economics, artificial intelligence and neuroscience, among others. Indeed, in recent years, Optimal Feedback Control theory has become quite popular in explaining certain aspects of human motor behavior [8], [9]. This kind of method results in feedback laws, which allow for closed loop control.

However, aside from certain classes of problems with a convenient formulation, such as the Linear Quadratic case and its extensions [10], or through linearization of the problem, achieved by adapting the immediate cost function [11], the exact total optimal solution in the discrete case is intractable due to the curse of dimensionality [1].

Thus, a lot of work in this field is devoted to find approximate solutions and efficient methods for computing them.

Heuristic search methods try to speed up optimal probabilistic planning by considering only a subset of the state space (e.g. knowing the starting point and considering only reachable states). These algorithms can provide offline optimal solutions for the considered subspace [12]–[14].

Monte-Carlo planning methods that doesn't manipulate probabilities explicitly have also proven very successful for dealing with problems with large state space [15], [16].

Some methods try to reduce the dimensionality of the problem in order to avoid memory explosion by mapping the state space to a smaller parameter space [17], [18] or decomposing it hierarchically [19]–[21].

Another family of approximation methods which has recently proven very successful [22] is the “determinization”. Indeed, transforming the probabilistic problem to a deterministic one optimizing another criterion allows the use of very efficient deterministic planner [23]–[25].

What we propose here is to do something rather different, by considering goal-independent distances between states. To compute the distance we propose a kind of determinization of the problem using a one step transition “mean cost per successful attempt” criterion, which can then be propagated by triangle inequality. The obtained distance function thus confers to the state space a quasi-metric structure that can be viewed as a Value function between all states. Theses distances can then be used to compute an offline policy using a gradient descent like method.

We show that in spite of being formally suboptimal (except for the deterministic and a described particular case), this method exhibits several good properties. We demonstrate the convergence of the method and the possibility to compute distances using standard deterministic shortest path algorithms. Comparison with the optimal solution is described for different classes of problems with a particular look at problems with *prisons*. Prisons or absorbing set of states have been recently shown to be difficult cases for state of the art methods [26] and we show how our method naturally deals with these cases.

## Materials and Methods

### Quasimetric

Let us consider a dynamic system described by its state 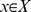 and 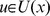, the action applied at state 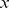 leading to an associated instantaneous cost 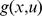. The dynamics can then be described by the Markov model: 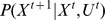
where the state of the system is a random variable 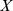 defined by a probability distribution. Assuming stationary dynamics, a function 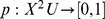 exists, satisfying 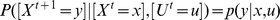



This model enables us to capture uncertainties in the knowledge of the system's dynamics, and can be used in the Markov Decision Process (MDP) formalism. The aim is to find the optimal policy 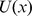 allowing a goal state to be reached with minimum cumulative cost. The classic method of solving this is to use dynamic programming to build an optimal Value function 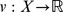, minimizing the total expected cumulative cost using Bellman equation: 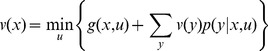
(1)which can be used to specify an optimal *control policy*

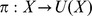


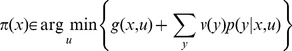
(2)


In general this method is related to a goal state or a discount factor.

Here we propose a different approach by defining a goal independent *quasimetric* structure in the state space, defining for each state couple a distance function 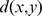 reflecting a minimum cumulative cost.

This distance has to verify the following properties: 
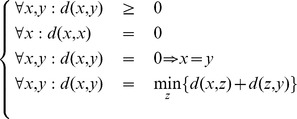
leading to the triangle inequality 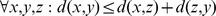



Therefore, the resulting quasi-distance function 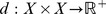 confers the property of a quasimetric space to 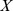.

Notice that this metric need not be symmetric (in general 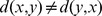). It is in fact a somewhat natural property, e.g. climbing stairs is (usually) harder than going down.

By then choosing the cost function 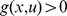 this distance can be computed iteratively (such as the Value function).

For a deterministic problem, we initialize with:
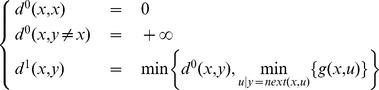
with 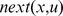 the discrete dynamic model giving the next state 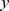 by applying action 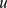 in state 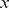. Then we apply the recurrence: 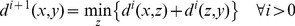
(3)


We can show that this recurrence is guaranteed to converge in finite time for a finite state-space problem.

Proof.

1. by recurrence 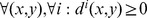 as:

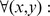

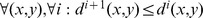
 as:

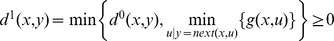
 as


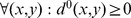
 and 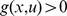 by definition.

- and if 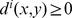 then
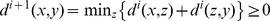

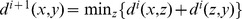
 then 
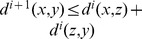
 in particular if we take 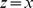 we have 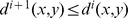.

3. 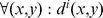 is a decreasing monotone sequence bounded by 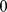.

However, finding a way to initialize 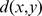 (more precisely 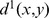) while taking uncertainty into account, presents a difficulty in probabilistic cases as we cannot use the cumulative expected cost like in Bellman equation.

For example we can choose: 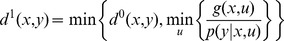
for the first iteration with 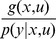 as the *one-step distance*.

The quotient of cost over transition probability is chosen as it provides an estimate of the *mean cost per successful attempt*. If we attempt 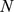 times the action 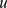 in state 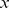 the cost will be 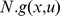 and the objective 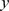 will be reached on average 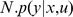 times. The mean cost per successful attempt is:
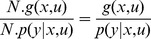



This choice of metric is therefore simple and fairly convenient. All the possible consequences of actions are clearly not taken into account here, thus inducing a huge computational gain but at the price of losing the optimality. In fact, we are looking at the minimum over actions of the *mean cost per successful attempt*, which can be viewed as using the best mean cost, disregarding unsuccessful attempts, i.e. neglecting the probability to move to an unwanted state.

In a one-step decision, this choice is a reasonable approximation of the optimal that takes both cost and probability into account.

This cost-probability quotient was used before to determinize probabilistic dynamics and extract plans [21], [27], [28]. Here we generalize this method to construct an entire metric in the state space using triangle inequality.

We also notice that contrary to the dynamic programming approach, the quasimetric is not linked to a specific goal but instead provides a distance between any state pair. Moreover, using this formalism, the instantaneous cost function 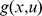 is also totally goal independent and can represent with greater ease any objective *physical* quantity, such as consumed energy. This interesting property allows for much more adaptive control since the goal can be changed without the need to recompute at all. As shown in the following, it is even possible to replace the goal state by a probability distribution over states. Another interesting property of the quasi-distance 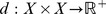 is that it doesn't have local minimum from the action point of view.

In fact, for any couple 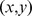, 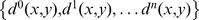 is a decreasing finite series of non-negative numbers (finite number of states), which therefore converges to a non-negative number 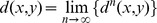



Note that if we multiply the cost function by any positive constant, the quasimetric is also multiplied by the same constant. This multiplication has no consequence on the structure of the state space and leaves the optimal policy unchanged, therefore we can choose a constant such that: 
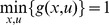



Let 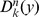 be the subset of 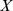 associated with a goal 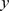 such that: 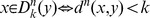
and let 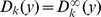 the subset of 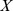 associated with the goal 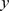 such that: 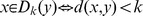



The subset 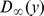 is the set of states from which the goal 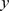 can be reached in a finite time with a finite cost. Starting from 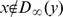 the goal 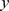 will never be reached either because some step between 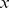 and 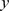 requires an action with an infinite cost, or because there is a transition probability equal to 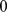.

Then the defined quasimetric admits no local minimum to a given goal in the sense that for a given 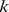, if 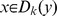 is such that: 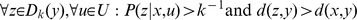
then 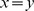


Proof.

1. if 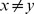 and 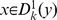, then 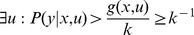 and

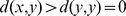
. As 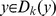 it is a counterexample of the definition.
2. if 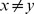 and 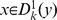, then 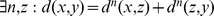. As 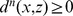, 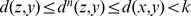, therefore 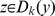.

- If 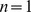, 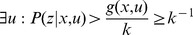 it is a counterexample of the definition.
- If 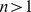, 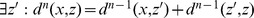. As 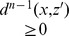 we have still 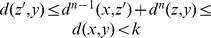 and therefore 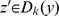.

– if 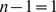 it is a counterexample.
– else we repeat the search for intermediary state. Thus by recurrence, there exists some state 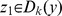 such that 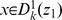 which gives a counterexample to the definition.

Consequently, if 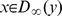, one can set 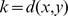 (a finite distance) and apply the above property to show that there exists at least one action 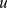 transforming the state 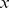 to some state 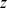 with a transition probability 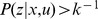 such that 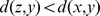.

### HMM case

In the real world, the state of the system is never really known. The only available knowledge we have consists of a series of observations reflecting *hidden* states. Probabilistic inference based on transition probability and observation likelihood allows to compute the probability distribution over the hidden states. This class of systems is usually modeled as Hidden Markov Models (HMM) and the problem of controlling such a system becomes a Partially Observable Markov Decision Process (POMDP).

Extending the quasimetric method to the POMDP case does not however, come without cost. Ideally, as with the theoretical POMDP, we should define a quasi-distance not just on the state space but on the belief space (estimated distribution over states), which is continuous and consequently difficult to deal with [29].

A possible approximation is to compute the policy not on the belief space, but on the observations-actions space, obtaining 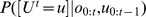.

Let us assume that we have a state observer maintaining a distribution over states, knowing all previous observations and actions 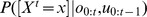.

At time 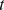 we know all the observations 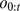 and all the previous actions 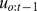, thus the distribution for the state can be recursively updated by the forward HMM equations: 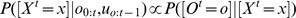


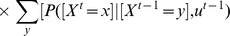


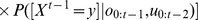
with 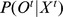 the observation model.

Then the distribution over action space can be computed by marginalizing over state space: 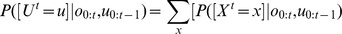


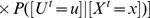
(4)assuming we have already computed the state dependent action policy 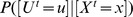 (see below).

Following this, a decision must be made based on this distribution. The chosen action can be random 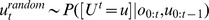
the most probable 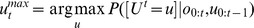



or the *mean*

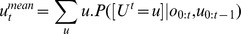



Here we assume a separation between state estimation and control, considerably reducing the computational cost compared to the optimal POMDP solution, which is intractable for most real-life problems.

One drawback however, is that the resulting policy could be less optimal and lacking in information-gathering behavior, for example.

### Probabilistic policy

As we have seen, in the classic MDP formalism, the policy 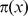 is a *deterministic* mapping of the state space 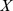 toward the action space 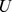 (using 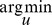). Pure MDP formalism only considers the optimal action (greedy policy), so a choice is made during the computation of the policy to only consider the one action that minimizes the cost.

However, this method could be viewed as arbitrary to a certain extent, especially for multimodal cases where the choice of a unique optimal action may lead to loss of information or blocking behavior.

In the field of reinforcement learning, the greedy policy is usually avoided in order to maintain exploratory behavior. To do so, methods such as 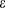-greedy, soft 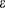-greedy and soft-max action selection were employed [7].

Here we propose building 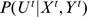 with 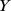 the goal, using a Gibbs distribution (soft-max like form):
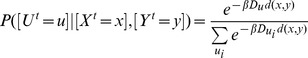
(5)with 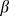 a parameter modulating the *sharpness* of the distribution (and consequently the exploration rate), and 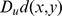 a *probabilistic gradient* of the quasi-distance:
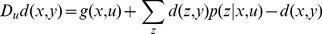
(6)


This gradient takes the immediate cost of the action into account, as well as the difference between the expected and current quasi-distances.

The resulting distribution depends on a goal 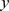, which can be fixed or even an evolving distribution 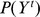. The latter distribution can represent multiple objectives or just uncertainty with respect to the goal. We can then obtain a the state dependent action policy by marginalizing:
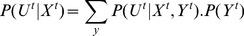
(7)


This way to build a policy can certainly be applied to any *potential*, such as the Bellman Value function. Similarly to reinforcement learning methods, actions are weighted according to their “value estimate” which, in our case, is the gradient of the expected quasi-distance. In MDP, the current state is known, so that the probability distribution over action space is directly given by the state dependent action policy (eq. 5 or eq. 7). In POMDP, the current state is not known, but by marginalization over the state space, one can also compute the distribution over the action space (eq. 4).

We can see that if 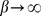, the policy tends toward a Dirac delta distribution (if a unique action minimizing the value exists). This extreme case reduces to the MDP optimal policy where a unique optimal action is mapped to each state, similarly to the Value Iteration or Policy Iteration methods.

The knowledge of a distribution on 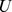 allows a random draw decision to be made from the distribution which can be useful to avoid blocking behavior or even learning. According to the 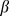 value, the soft-max policy associated with the random draw decision generates either a more optimal behavior (large 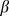) or a more exploratory behavior (small 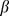).

## Results and Discussion

### Comparison with dynamic programming

#### Convergence and complexity

First as we have shown, computation of the quasi-distance is ensured to converge even for infinite horizon (in finite time for a finite state space) while the standard Value Iteration algorithm is not. In fact, it is usually necessary to introduce a *discount factor* 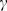 in Bellman's equation to assure convergence, but at the price of sub-optimality whereas such a thing is not needed with the quasimetric.

Constructing the initial *one-step distance* 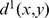 for all state couples is in 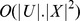. Then, directly applying the recurrence in equation 3 leads to a complexity of 
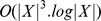
 for the whole state space (all-to-all states). However, the quasimetric construction uses probabilities only at the first iteration (i.e. the *one-step distance*) and then propagates these distances with triangle inequality. This propagation of *one-step distances* is completely deterministic and no more probabilities appear afterward. Thus, computing the quasi-distance can be reformulated in a graph theory framework as a deterministic shortest path problem.

Let us consider the weighted directed graph (or network) 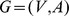 with vertices 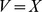 and arcs 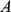 the set of ordered pairs of vertices. We can assign to each oriented arc 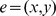 the weight 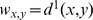 (the *one-step distance*). Remark that for the sake of efficiency it is preferable to consider an arc only if the associated weight 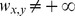, i.e. if an action 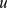 exists with a finite cost and for which the transition probability 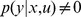. Constructing this graph is of the same complexity than the 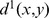 iteration and is only computed once nonetheless.

Then, the problem of computing the quasi-distance from 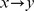 becomes the problem of finding the length of the shortest path between vertices 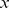 and 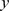.

One can compute the whole quasimetric (all-to-all states) by computing the all-pairs shortest paths using for example the Floyd-Warshall algorithm [30]–[32]. However, considering the usual MDP problem with a fixed goal, one would prefer to compute the quasi-distance for only one goal, which can be viewed as the multiple-source shortest path. An efficient way to solve this is to consider the transposed graph 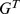 in which arcs are inverted and to solve the single-source shortest path (from goal vertex) using for example Dijkstra's algorithm [33] or 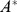 depending on the problem [34].

From a computational point of view, using Dijkstra's algorithm to solve the one-goal problem can be done with the 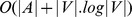 worst case complexity using the appropriate data structure [35]. Knowing that 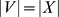 and 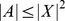 it's 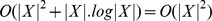, considering a fully connected graph.

This is to be compared to classical discounted Value Iteration method which complexity is 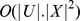 for one iteration (or sweep) with the worst case number of iterations to converge proportional to 
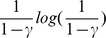
, 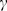 being the discount factor [36].

Notice that, transition probabilities are usually sparse allowing the graph to be equally sparse. Hence, considering the mean vertex out-degree 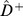, complexity using Dijkstra's method becomes 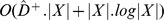, 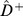 depending on the dispersion of transition probabilities.

Therefore, quasi-distance can then be easily solved in a computationally efficient way using the standard deterministic graph theory methods.

#### Equivalence

The question is, how much does the quasimetric method diverge from the dynamic programming? In other words, how can we compare the quasi-distance with the value function in order to discuss the optimality approximation? In order to be able to compare, we first have to consider only a subset of the quasimetric by looking at the quasi-distances from all states to one unique state (a goal). If the quasi-distance and the value function are equal for a specific goal (strong equivalence) then clearly policies obtained with both methods will lead to the same behavior. But it is also possible that the quasi-distance differs from the value function and still yield the same policy (weak equivalence).

In the deterministic case, the quasi-distance and the value function are trivially equal. But there is at least one other class of problems where these two approaches are strictly equivalent that we call the *probabilistic maze*.

Let us consider a probabilistic system where the uncertainties concern the success of actions. If an action 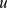 succeeds, it drives the system from one state 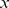 to another state 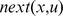; if it fails, the system remains in state 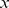. We can then call 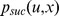 the probability that action 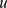 is successful from state 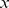. This function determines all the transition probabilities that are null except for:
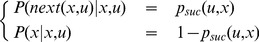
(8)


This kind of systems was also described as “self-loop MDPs” and used for MDPs determinization [27]. For this class of systems – which includes those that are deterministic – the value function and the quasi-distance are strictly equivalent and lead to the same optimal policy.

Indeed, we can inject these probabilities in the Bellman's equation: 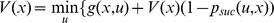


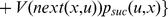
(9)


So 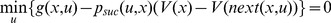
(10)


In each state 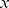 there exists at least one optimal action 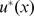 such that: 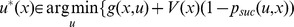


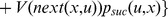
(11)


In a probabilistic maze, an action can only succeed – thus driving the system from 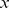 to 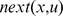 – or fail and leaving the system unchanged. So starting from any initial state 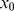, the optimal policy 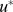 describes a unique optimal trajectory 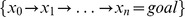. If the optimal action fails in some state 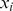, the system remains in 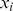 and the optimal action to apply is still the same. So 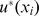 is repeatedly chosen until it succeeds to move the system to 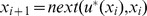 and the probability to succeed in exactly 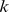 tries is 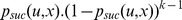. Therefore, the mean cost for the transition 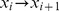 is:
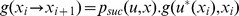


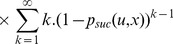
(12)


and as 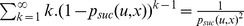 we have: 
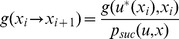
(13)


So the optimal policy is the one which minimizes this mean cost per successful attempt 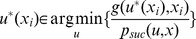
(14)


Finally we have: 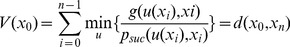
(15)


**Figure 1:**
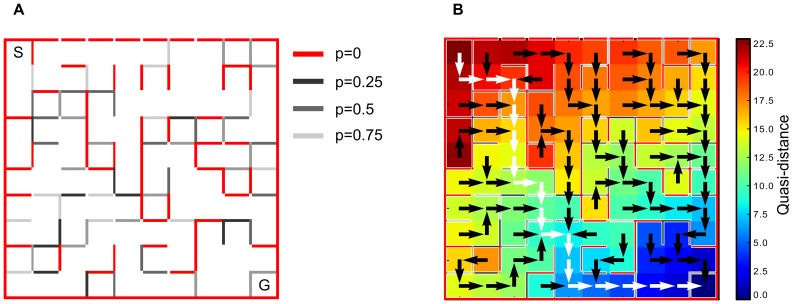
Example of a simple *probabilistic* maze of size 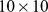 where dynamic programming and quasimetric methods are equivalent. (A) S is the starting state and G the goal. The red wall cannot be traversed (transition probability 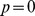) while gray ones can be considered as *probabilistic doors* with transition probability 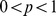. 5 actions are considered: not moving, going east, west, south and north. (B) Quasi-distance obtained for the probabilistic maze example with a constant cost function (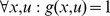) and corresponding policy. White arrows represent the optimal policy from position S to G. Black arrows represent the optimal policy to reach G from other positions.

Figure 1 shows an example of such a maze, with the corresponding quasi-distance and policy.

This type of problem may appear somewhat artificial but it can for example, refer to a *compressed* modeling of a deterministic system, exploiting the *structure* of the state space.

Let us imagine a mobile agent in a corridor. In a discretized space, the corridor can have length 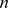, each action moving the mobile one cell forward with the probability 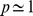.

In order to exit the corridor, the action has to be applied 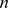 times. Alternatively, this discrete space can be *compressed* by representing the corridor with a single cell and the probability to succeed (i.e. to exit the corridor) 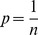. The resulting model is the probabilistic maze described above.

#### Non-equivalence

In the general case, the quasimetric approach will differ from the dynamic programming method. These differences arise when the transition probabilities are *spread out* along several arrival states. This dispersion of arrival states can produce differences between the quasi-distance and the value function with – or without – differences in the optimal policy obtained.

#### Systems yielding a quasi-distance different to the value function

**Figure 2:**
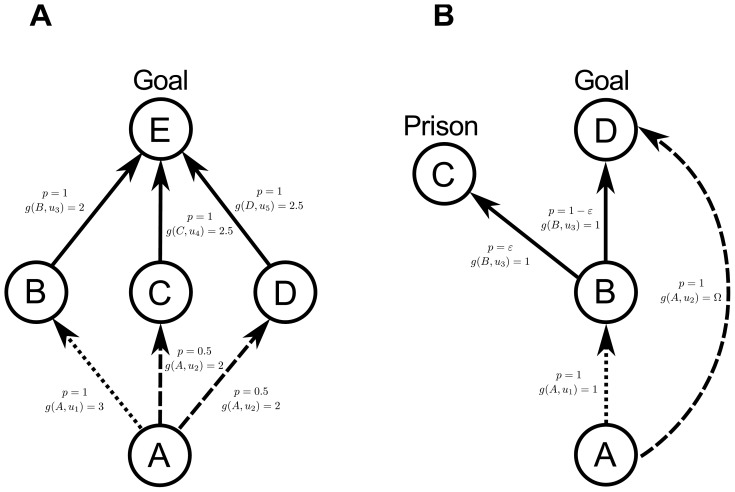
Simple systems where the quasimetric and the dynamic programming methods are not equivalent. (A) Example of non deterministic systems where the quasi-distance differs from the value function.Arrows indicate possible actions with their associated transition probabilities 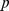 and costs. Dotted arrow represents action 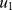 and dashed arrows action 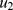, both allowed in state 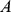. (B) Example with a prison state 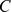. Starting from 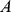 to the goal 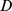 we can choose between two actions. Action 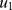 in dotted leads to 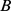 with a low cost but then with the risk to fall from 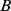 to 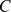 with a probability 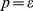. 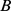 is a risky state. Action 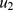 in dashed leads to the goal with a probability 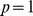 but with a high cost 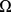.

Here is a simple case illustrating the difference between the two methods. Let us consider a system with fives states 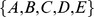 where 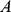 is the starting state and 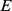 the goal (cf. Fig. 2A). This system is almost deterministic since the only uncertainty relates to one action in state 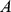. For 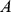 there are two possible actions, one driving the transition 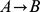 with a probability of 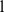 and a cost of 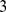 (action 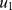) and one driving either 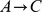 or 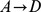 with a probability of 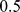 and a cost of 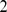 (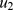). Then from 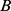, 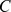 and 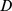 the transition are deterministic, with associated costs of respectively 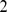, 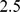 and 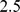.

The corresponding computed quasi-distances can be found in table 1. The shortest path according to the quasi-distance is 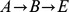. The optimal policy in 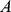 however, is to choose the action 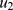 leading to either 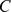 or 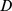 with a probability of 0.5 and a cost of 2. Indeed, for the action 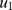 leading to 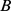 we have 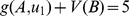 whereas 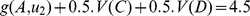.

**Table 1:** Quasi-distances and Value function for example 2A.

	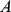	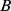	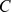	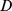	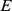	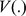
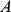	0	3	4	4	5	4.5
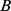	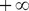	0	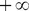	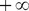	2	2
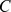	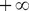	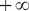	0	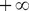	2.5	2.5
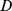	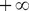	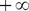	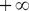	0	2.5	2.5
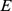	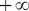	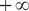	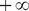	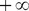	0	0

The value function of state 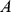 is slightly lower than 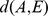 but both methods lead to the same optimal choice of 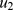 while in 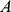.

In this example, the quasi-distance yields an inaccurate estimate of the mean cost when starting from state 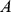. In fact, the quasi-distance computation tends to favor actions with low dispersion in transition probabilities (low uncertainty). So here, the quasi-distance obtained differs from the Value function for state 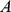, but generates the same optimal policy.

The policy can also differ in the general case. In fact, replacing all the costs in the same example with 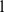 leads to 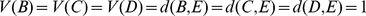. However, due to the uncertainty of action 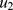 we have 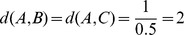 and 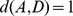, thus clearly biasing the policy obtained with the quasi-distance in favor of 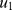. On the contrary, as the Value function takes all of the consequences of actions into account, 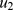 leads to 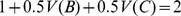 and 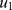 to 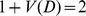, so the two actions are equivalent. Roughly speaking, the quasi-distance yields an uncertainty aversive policy, resulting from the 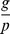 form of the one-step distance.

#### Systems with *prison-like* states

Control under uncertainty can be viewed as a continuous decision making where both cost and uncertainty must be dealt with. The trade-off between cost and uncertainty can be illustrated by the spider problem [37] where an agent can reach a goal quickly by crossing a narrow bridge or by slowly walking around a lake. In a deterministic case, crossing the bridge is the obvious optimal action, but when there is uncertainty as to whether the spider is able to cross the narrow bridge, the optimal action could be to walk safely around the lake (as falling into the water may be fatal).

As regards the spider problem, falling into the water may bear a sufficiently large cumulative cost to justify choosing to walk around the lake hazard-free. But then, what happens when confronted with a choice between a very costly but certain action and a low-cost action where there is a small probability of death? Clearly this problem may be much more difficult as death may not be associated with a high cost per se. The action of “walking” when in state “bridge” has no objective reason to be higher than “walking” when in state “lakeside” if we consider energy consumption. Instead, the problem with death does not lie in the cost but in the fact that it is an irreversible state.

In our modeling, death can be represented as a *prison* state from which one cannot escape. So this singularity is slightly different from a state-action with a high cost. In general these prisons can be a subset of the state space rather than a unique state. These particular states, sometimes referred as “dead-ends”, are known to be problematic for MDPs and are implicitly excluded from the standard definition as their existence may prevent solution to converge [38]. Moreover, recent works in the domain of planning have identified these problems as interesting and difficult, recognizing the need to find specific methods to deal with it [22]. It is to be noted that a prison is an absorbing set of states that are not necessarily absorbing as it may be possible to “move” inside the prison. In fact a prison is as a set of states that does not contain the goal and from which we cannot reach the goal.

To illustrate this class of problems, let us imagine that the spider is unable to swim. Falling into the water now leads to a prison state (death of the spider).

Figure 2B models this problem considering four states 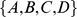 where 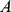 is the initial state and 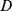 the goal. There is a choice of actions in state 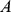. The first action 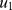 drives the transition 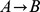 with a probability 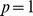 and a cost of 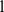. Then from 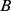, the unique action can lead to 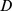 with 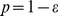 or 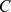 with a probability 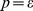 and a cost of 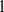. The second action 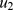 allows for a transition 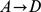 with a probability 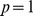 but a cost 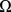.

In this case what should the spider do? The computed value function and the quasi-distance can be found in table 2. According to Bellman's equations, the action 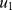 should never be attempted. Indeed, for state 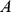 and action 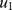 we have 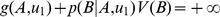 and for 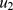 we have 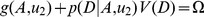. So clearly the optimal choice here according to the value function is 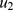, independently of cost 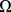 which can be seen as rather radical.

**Table 2:** Quasi-distances and Value function for example 2B.

	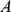	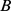	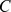	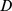	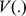
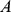	0	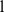	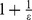	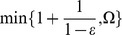	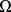
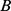	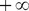	0	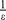	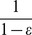	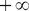
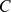	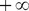	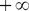	0	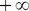	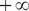
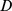	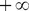	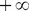	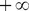	0	0

In contrast, the action policy obtained with the quasi-distance depends on the relative values of 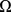 and 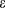 (cost vs. uncertainty) that are parameters of the problem. Indeed, 
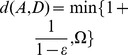
 involves:
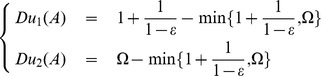



So if 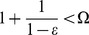 we have 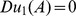 and 
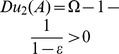
 then 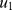 is chosen.

But if 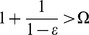, 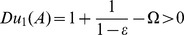 and 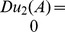, then 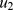 is chosen.

We see that different policies can be chosen depending on the problem whereas dynamic programming will always avoid 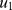. It is then also possible to modify the cost function in order to move the cursor of the risky behavior by changing the cost of 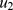.

We can formalize these *prison-like* states further in order to better control these effects.

Let us define state 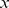 as belonging to the prison 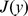 of state 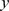 if there is no policy allowing the transition from 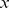 to 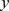 with a non zero probability.

We notice that if we compute the reaching set 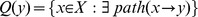, we can obtain 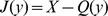. Then, by definition 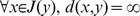.

With our method, these prison states are states for which the quasi-distance to a specific (goal) state is infinite. Moreover, contrary to dynamic programming methods, for a finite cost function (and in a finite state space) the prison states are the only states with infinite quasi-distance to the goal, making them easy to identify. In fact, as described, we initialize all distances with: 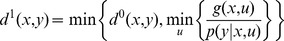



So any “one-step” distance between two states 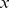 and 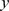 will be finite if at least one action 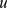 with a non zero probability 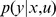 exists. Then, these “one-step” distances are propagated by triangle inequality ensuring that 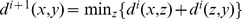 is infinite iff the probability of reaching 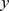 from 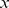 is zero, i.e. there is no path between 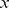 and 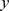. Thus with our method, considering a finite cost function and a finite state space, every prison state has an infinite distance and every state with infinite distance is a prison state.

As described, the proposed general quasi-metric iterative algorithm can detect all the possible prison states and for a goal directed MDP, the proposed deterministic shortest path algorithm for computing the quasi-distance will also naturally detect these prisons without propagating to other states.

Furthermore, there are also *risky* states that do not belong to 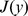 but are still associated with an infinite Value function. Obviously, all states in 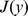 have an infinite Value but contrary to the quasimetric the reciprocal is not true. Therefore, all states with a non zero probability of leading to a prison state also have an infinite Value (propagated by the conditional expectation in Bellman's equation): 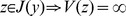
so we have
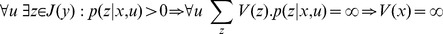



Thus the infinite value can propagate to the whole state space depending on the distributions. This property of the dynamic programming method makes prison states indistinguishable from other risky states if there is no “complete proper policy” (a policy leading to the goal with a probability of 1). This may also prevent any policy to be computed. Indeed, all the possible policy can appear to be equivalent when all states have an infinite Value. This case appears if we remove the action 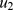 in this example, then the prison becomes “unavoidable”. In recent years, a number of work have been devoted to formalizing, detecting and dealing with these prisons in the planning domain, in particular for “unavoidable” ones [22], [26], [39], [40]. The straightforward method we propose here allows for a finer grained management of this risk.

The set 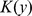 of these risky states can be constructed iteratively or by looking at 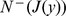, the set of predecessors of 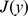. We can observe that 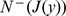 is the set of states for which at least one action leads to a prison state with 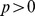. We call this set the *weakly risky* states 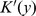: 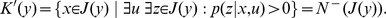



The risky set is: 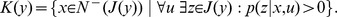



We can even decide a minimal acceptable risk 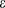 (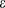-risky set), such as: 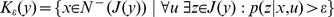



As seen in the previous example, from a quasi-distance point of view we can consider a risky state to be very close to the objective. If in a particular state 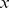 all actions carry the probability 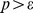 of entering a prison state, but at least one (let's say 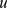) also has 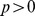 of going directly to the objective 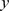, we have 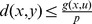. In order to avoid these risky states it can be decided that 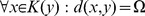 with 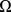 an arbitrary large value (possibly infinite).

This ability to deal with risk contrasts with the classic dynamic programming method, according to which one should *never* cross the road or use a car, considering that there is always a non zero probability of an unavoidable and irremediable accident (prison state). However, by crossing the road or using a car we put ourselves in a risky state, but not in a prison state!

Consequently, a distinction between 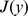 and 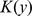 along with the ability to parametrize the risk/cost trade-off enabled by the quasimetric approach is essential and may be interesting for modeling human behavior.

### Applications

#### Under-actuated pendulum

Let us consider an under-actuated pendulum driven by the following equation:
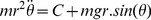
(16)with 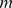 the mass, 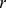 the radius, 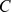 the torque, 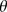 the angular position and 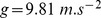.

The problem is to reach and maintain the unstable equilibrium 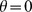 (upward vertical) from the starting stable one 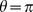 (downward vertical) with a minimum cumulative cost, knowing that we can only apply a torque 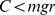. If we use as the time unit 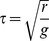, the time constant of the pendulum, we can reduce equation 16 to the dimensionless one:
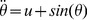
(17)with the normalized torque 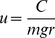 such as 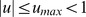.

Then, by considering 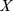, 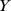 and 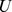 as the discrete variables, representing respectively 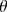, 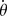 and 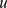, we can decompose the probabilities as follows:
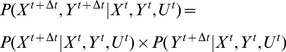
(18)and the following discrete Gaussian forms: 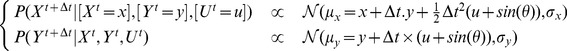
(19)with 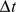 the discrete time step.

So here, we approximate in discrete time the equations of the dynamics with a Gaussian uncertainty hypothesis, described by parameters 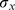 and 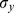.

Simulations were done for a state space of 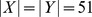 and 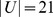 with 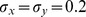.

**Figure 3:**
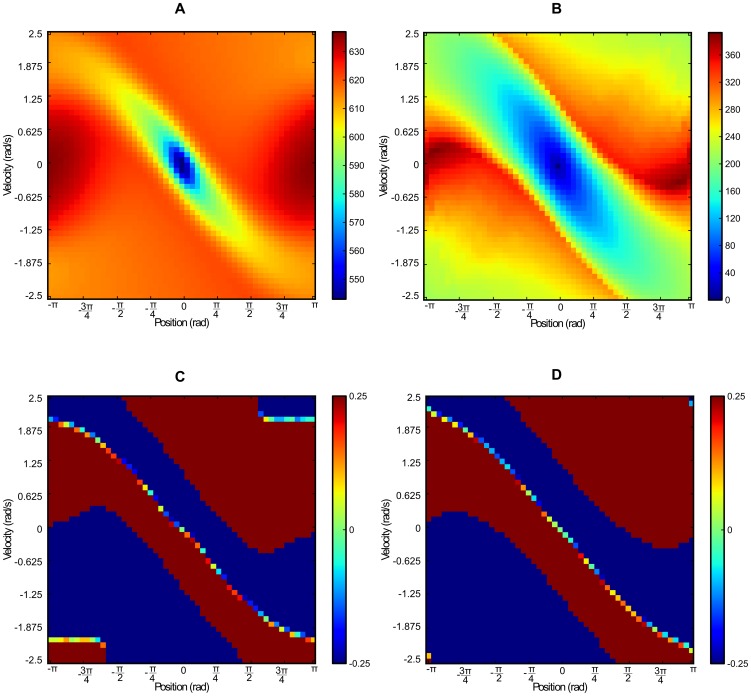
Comparison of obtained value function, quasi-distance and policies for the inverted pendulum system. (A) Value function obtained with undiscounted value iteration. (B) quasi-distance. (C) Policy obtained with undiscounted value iteration. (D) Policy obtained with the quasi-distance (most probable policy).

Using the following cost function: 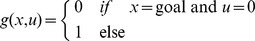
the obtained Value function and quasi-distance are similar but not equal (cf. Fig. 3).

For the Value function (without discount factor), the zero cost for the goal state (needed for convergence) only propagates very slightly and distant states have almost the same expected cost.

In contrast, the quasi-distance exhibits larger variations over states because it is not smoothed by the computation of the mean cost expectation of the Value Iteration method.

The constant cost chosen in this problem results in minimizing “path length” (number of state transition) and uncertainty (as the quasi-distance results in 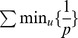).

We computed the optimal policy with Value Iteration: 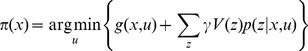



Similarly for the quasi-metric method we computed the argmin policy as the policy minimizing the expected distance:
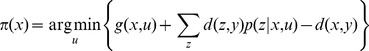



Figure 3 shows the deterministic policies obtained for both dynamic programming and quasi-distance. Here again, small differences occur even though the policies are mostly *bang-bang*. We notice that small differences also occur due to the border effect that is provoked by discretization.

Despite these differences in both the Value function and the policy, overall behavior is very similar.

**Figure 4:**
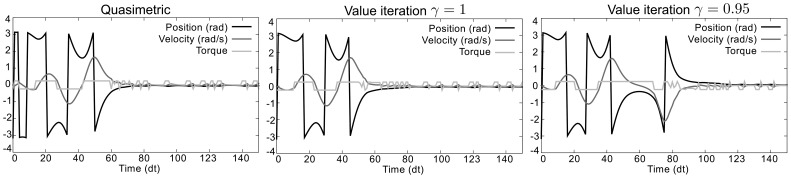
Behaviors obtained with quasimetric and dynamic programming methods with different discount factors. Starting from the initial stable state, both methods lead to the objective but with different trajectories.

Results of these policies can be seen on figure 4, starting from position 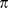 with a velocity of 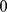. We can see that the trajectory obtained with the quasimetric method is slightly longer than that obtained with undiscounted Value iteration (optimal), but still better than that obtained with discounted Value iteration (suboptimal with 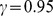).

**Figure 5:**
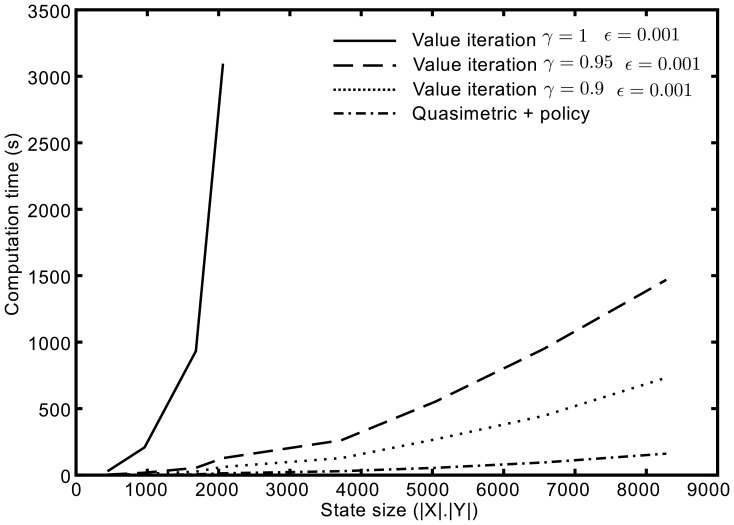
Comparison of computation time for the under-actuated pendulum example.

We also compared computation time in terms of state space size (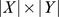 with a constant action space size 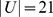) between discounted Value iteration and quasimetric methods. Figure 5 shows the results obtained for the single-goal quasi-distance (quasi-distance from all states to the goal) and its associated 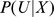 policy, along with Value iteration with different discount factors. These results were computed based on the same input transition probabilities with single thread C++ implementations of algorithms on an Intel Core2 Duo E6700 @ 2.66GHz desktop computer.

We can see that Value iteration heavily depends on the chosen discount factor and is much slower than the quasimetric method for discounts close to 1. Notice that computation time for the quasimetric method includes graph construction (which should be done only once), quasi-distance and policy. As an illustration, for a state space of 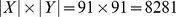, graph construction takes 129 s, quasi-distance (Dijkstra shortest path) takes 3 s and policy takes 31 s while Value iteration with 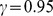 takes 1469 s.

#### Nonholonomic system

A slightly more complicated system is the Dubins car model [4]. This system is interesting because it exhibits nonholonomic constraints for which optimal control is difficult. However, it has generated a large amount of work during last decades and several studies have provided in-depth understanding and formal solutions of such systems and successfully applied optimal methods for real-world robots (see [42], [43]).

A Dubins car nonholonomic system is described with: 
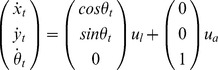
(20)with the control input 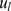 and 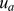 respectively the linear and angular velocity.

For the sake of simplicity, we constrain the linear velocity to a constant value 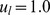 and the angular velocity 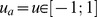.

If we consider a probabilistic version of this system, the transition probabilities for the dynamic model are:
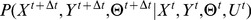
(21)which can be rewritten with some independence assumptions as the product: 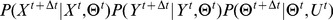
(22)and the following discrete Gaussian forms:
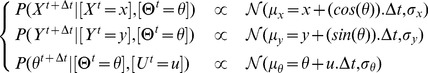
(23)


We computed the quasimetric for this system in a discretized state-space with the following parameters:
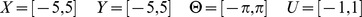


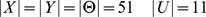


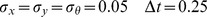



**Figure 6:**
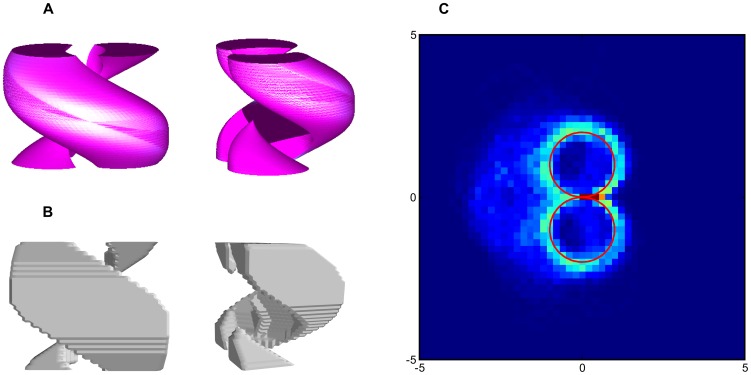
Results obtained for the non-holonomic system. (A) Accessibility volume of the Dubins car obtained with geometrical methods (adapted from [42]). (B) Discrete accessibility volume obtained for the described probabilistic case using the quasimetric method (axes aligned similarly). (C) Average trajectory for 500 simulations of 50 timesteps obtained starting at 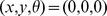 with goal at 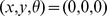 with a stochastic simulation and a drawn policy. Red curves are the optimal trajectories for a deterministic system.

The accessibility volume obtained from the origin – i.e. the volume of the state space that can be reached with a path length inferior to a given value – is very similar to the optimal deterministic one from [42] (cf. Fig. 6). Although it is not clear what signification accessibility volume could have for a probabilistic model, these two methods behave quite similarly. It is to be noted that this result was obtained with our general method without any specificity about the problem.

If we simulate this system by adding noise to the position, according to the described model, and use a random draw policy as described before we can see that the average trajectories obtained correspond mostly to direct loops (cf. Fig. 6).

These two symmetrical loops are comparable to the optimal deterministic behavior. On average, the behavior of the system closely match the theoretical deterministic optimal. Due to the noise, it is almost impossible to reach the goal in one trial. Most of the time the goal is missed (with one trajectory passing very close) and then the controller starts another loop or a figure of eight (trajectories on the left side of the red curves).

## Conclusion

We propose a new general method for control or decision making under uncertainty. This method applies for the discrete MPD case with positive cost and infinite or indefinite horizon. The principle of this approach is to define a goal independent quasimetric to the state space which can then be used to compute a policy for a chosen goal or set of goals. Thus, each distances from all to one state describing a subspace of the whole quasimetric can be viewed as an approximation of the Value function.

To compute the distances between states we proposed to used the “mean cost per successful attempt” of a direct transition that we propagate by triangle inequality. We show that this distance computation can be reformulated as a standard deterministic shortest path problem allowing the use of efficient algorithms. Thanks to this property we have shown that the quasimetric approach may lead to a very significant gain in terms of computational cost compared to dynamic programming. Illustrative examples were treated and have shown very good results.

We have demonstrated that for systems with possible prison states (excluding the goal), the quasimetric can significantly differ from the optimal solution when prisons are “avoidable”. Moreover this method is still able to produce a solution for problems with “unavoidable” prisons where standard dynamic programming approach cannot. We proposed a way to finely tune risk sensitivity and risk/cost trade-off, defining risky states and a possible threshold on risk taking. Interestingly, this kind of risk-related behavior is reminiscent of that present in humans and is still to be compared to classic methods in human decision making.

We also proposed a soft-max like way to compute a policy, which provides an entire distribution rather than a unique optimal deterministic action. Dealing with a probability distribution over actions provides, in our sense, a less restrictive way of considering control under uncertainty. With this method it is for instance, possible to make a decision when faced with multiple equivalent actions, thus introducing variability in actions and allowing exploration. This soft-max method, along with the random draw action, is also applicable to the Value function.

Extending this method to the HMM cases we described, is computationally very cheap compared to the optimal POMDP, which is usually intractable. Moreover, one can question whether solving the POMDP is relevant when the model is imperfect or may change over time. Although our method is not optimal in the general case and lacks information-gathering behavior, we think it could be a useful bootstrap for learning using available prior knowledge, even if the latter is very coarse. Indeed, it could occasionally be more interesting to use a simple model with uncertainties than a very complicated model which is nonetheless rarely perfect. Therefore, we could consider our method as a trade-off between solving the POMDP and learning from scratch.

Finally, this very general approach can be applied to a wide range of problems involving control under uncertainty. Although it is currently restricted to discrete space, infinite/indefinite horizon cases, we hope to see contributions from the community of control and planning as much of the techniques developed for dynamic programming can be applied to this method.
